# Supplementary figures and images for: Developmental Constraints on Vertebrate Genome Evolution
Source: PLoS Genet. 2008 Dec 19;4(12):e1000311. doi: 10.1371/journal.pgen.1000311 (PMC2600815; doi:10.1371/journal.pgen.1000311)

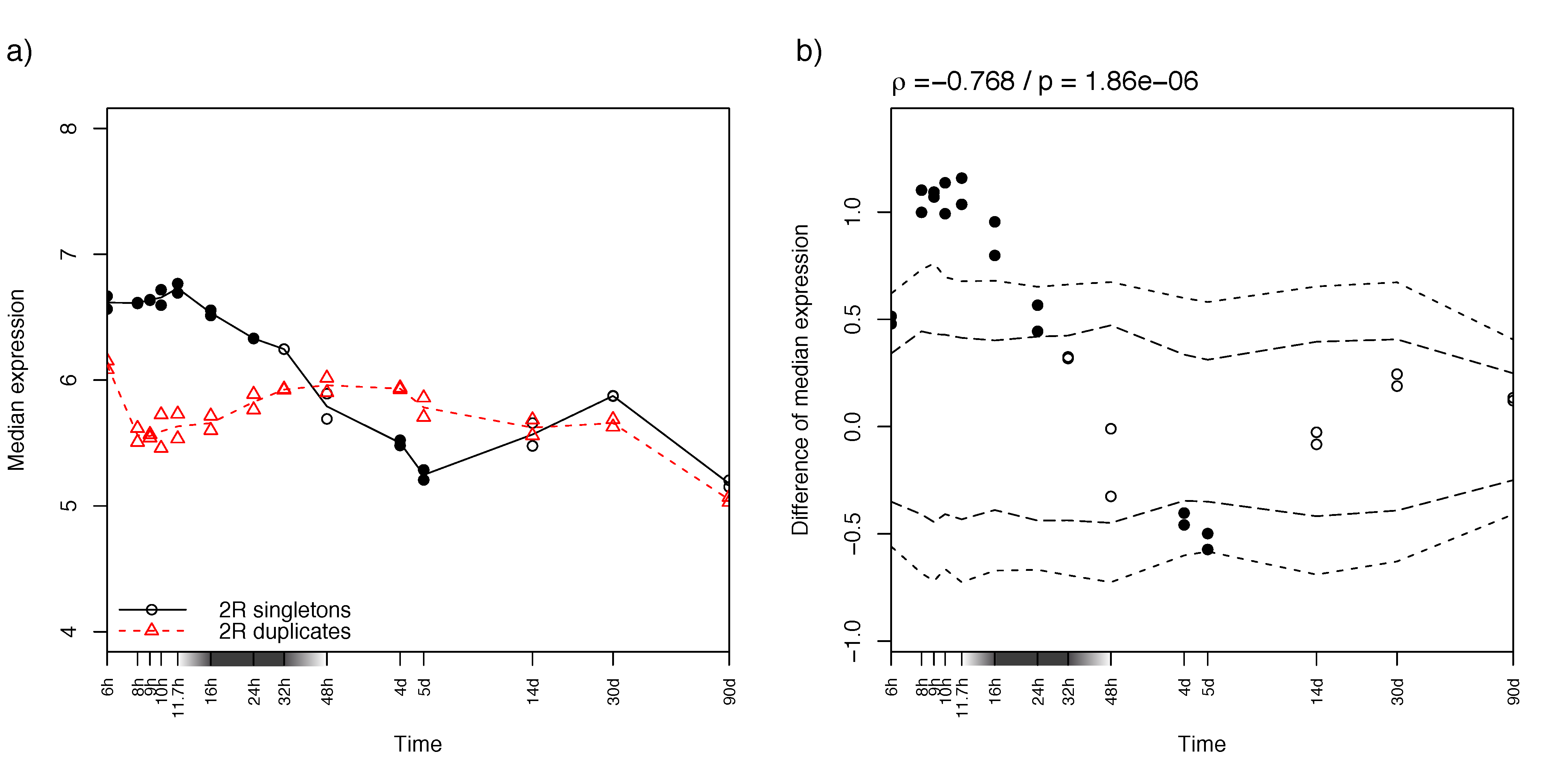

Supplement: Figure S1 — Expression in zebrafish development of genes according to retention after vertebrate 2R whole genome duplications. Median expression profiles of vertebrate specific 2R duplicates in zebrafish in red dashed line and triangles, and of singletons in black solid line and circles. Legend as in Figure 2. (0.53 MB TIF) [file pgen.1000311.s001.tif]

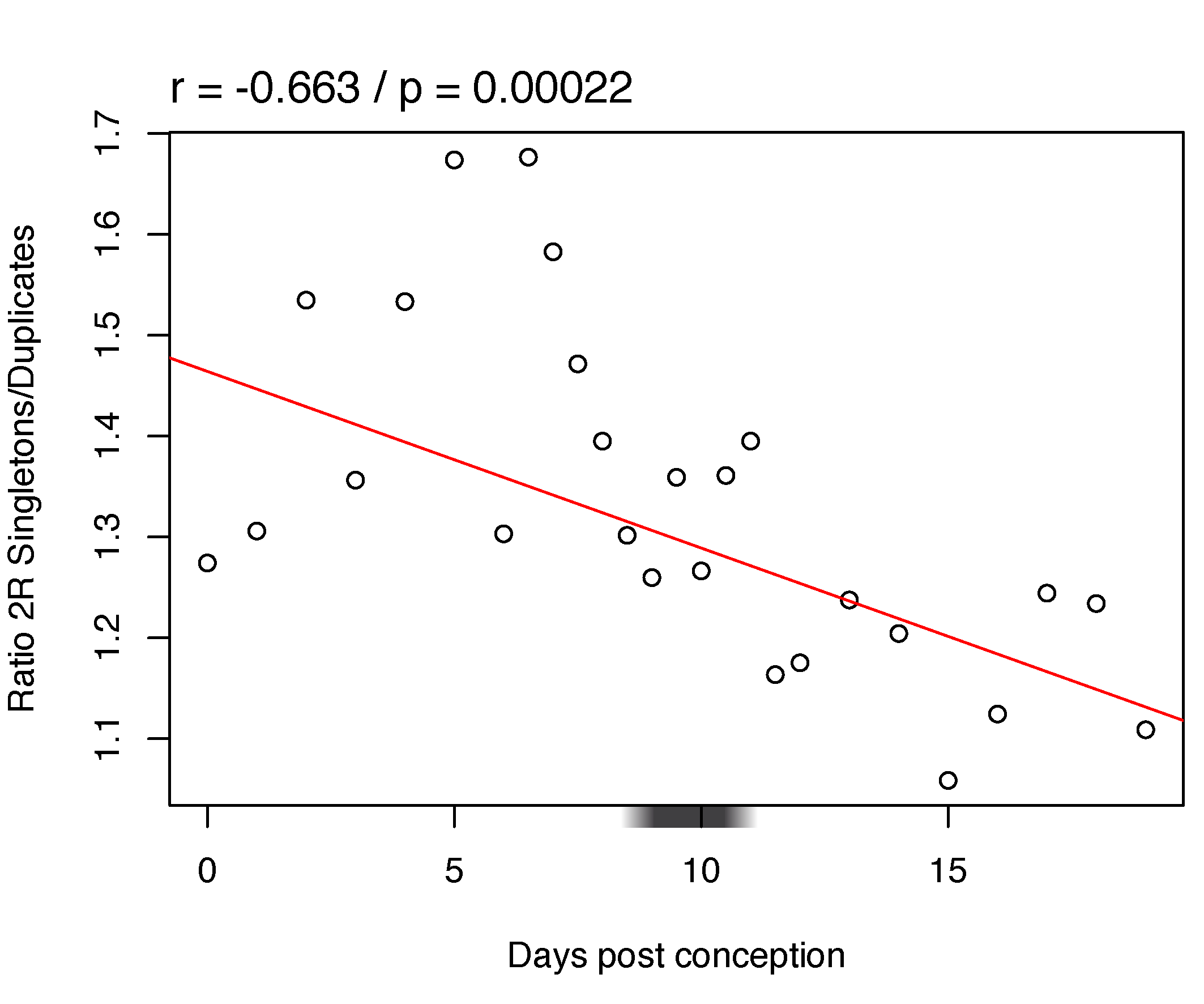

Supplement: Figure S2 — Variation across mouse development of the ratio of expressed vertebrate 2R singletons, relative to duplicates. Legend as in Figure 4. (0.31 MB TIF) [file pgen.1000311.s002.tif]

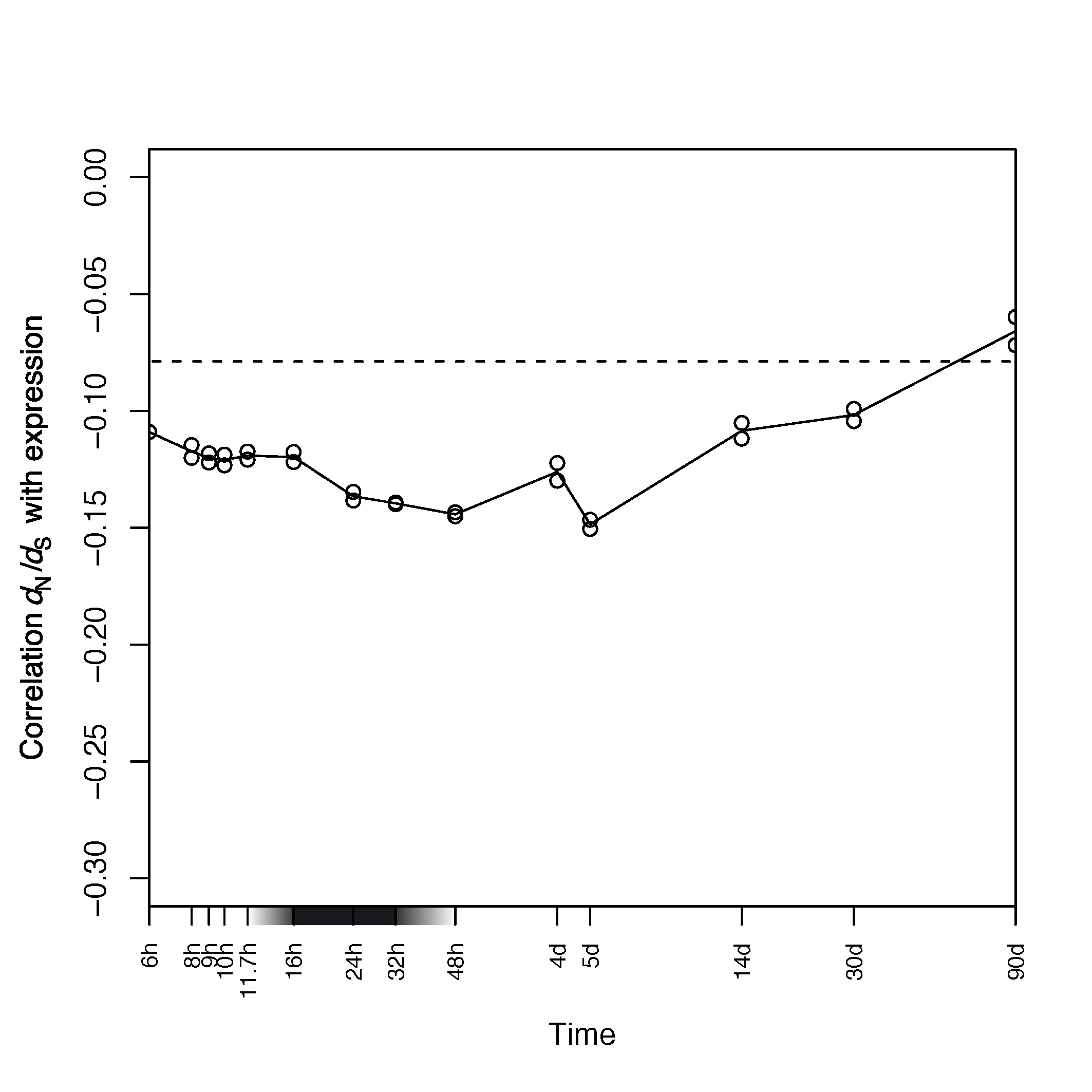

Supplement: Figure S3 — Variation across zebrafish development of the Spearman correlation between gene sequence evolution and expression. Only singletons genes (for 2R and fish-specific genome duplications) were considered. We used the ratio of the rate of non-synonymous substitutions on the rate of synonymous substitutions (d N/d S) as a measure of selective pressure. Correlations below the dashed line are significantly different from 0 (p-value <0.05). The x-axis is in logarithmic scale. A gray box on the x-axis indicates the phylotypic period. (0.41 MB TIF) [file pgen.1000311.s003.tif]

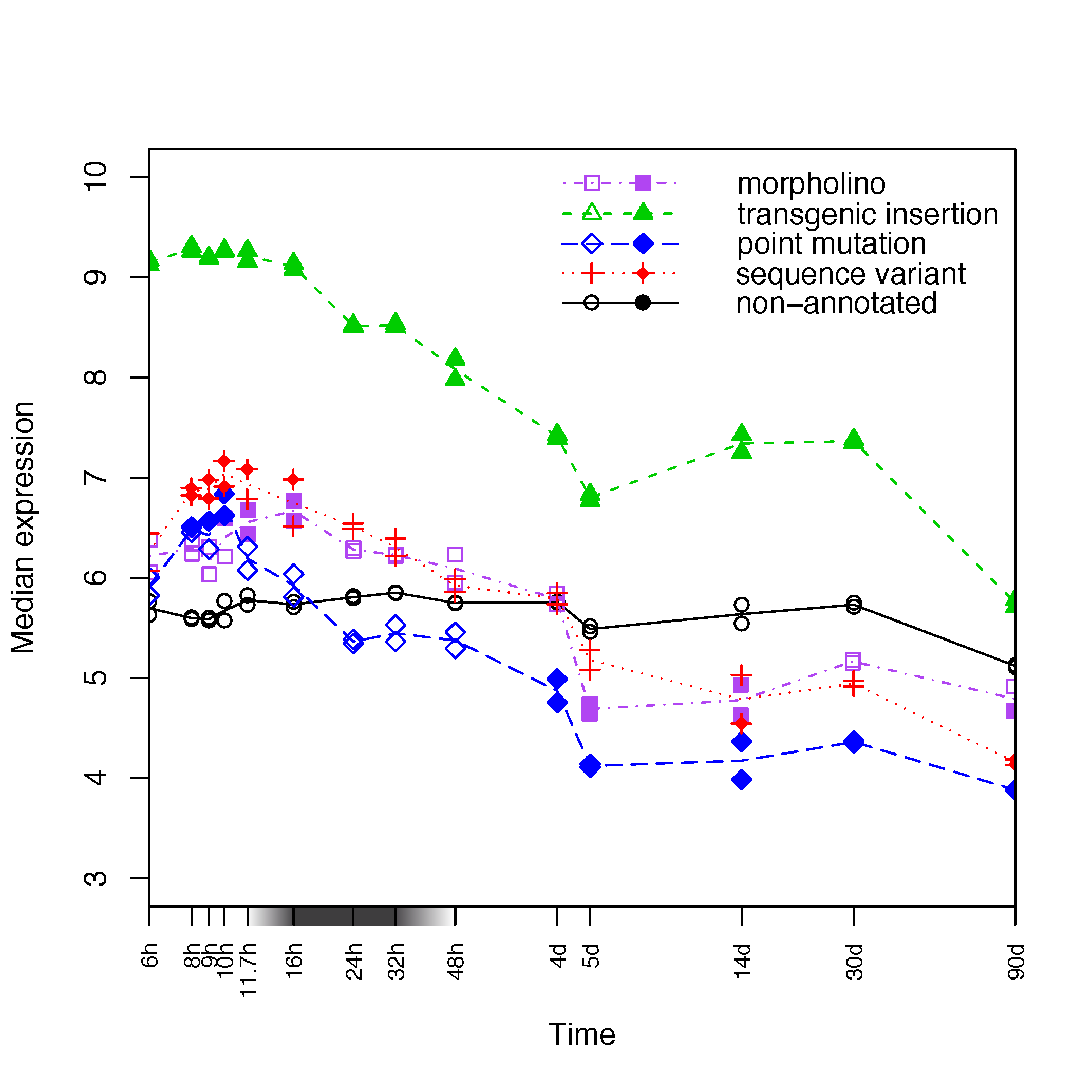

Supplement: Figure S4 — Expression in zebrafish development of genes with abnormal mutant phenotypes. Median expression profiles of zebrafish genes inducing abnormal phenotypes when non functional, for 4 different techniques, compared to non-annotated genes in black solid line and circles. The techniques are: morpholinos in purple dashed-dotted line and squares; transgenic insertions in green dashed line and triangles; point mutations in blue dashed line and diamonds; sequence variants in red dotted line and crosses. Points significantly different from the reference curve (non annotated genes) are filled. See Figure S5 for confidence intervals of the difference with the reference curve. The x-axis is in logarithmic scale. A gray box on the x-axis indicates the phylotypic period. (0.41 MB TIF) [file pgen.1000311.s004.tif]

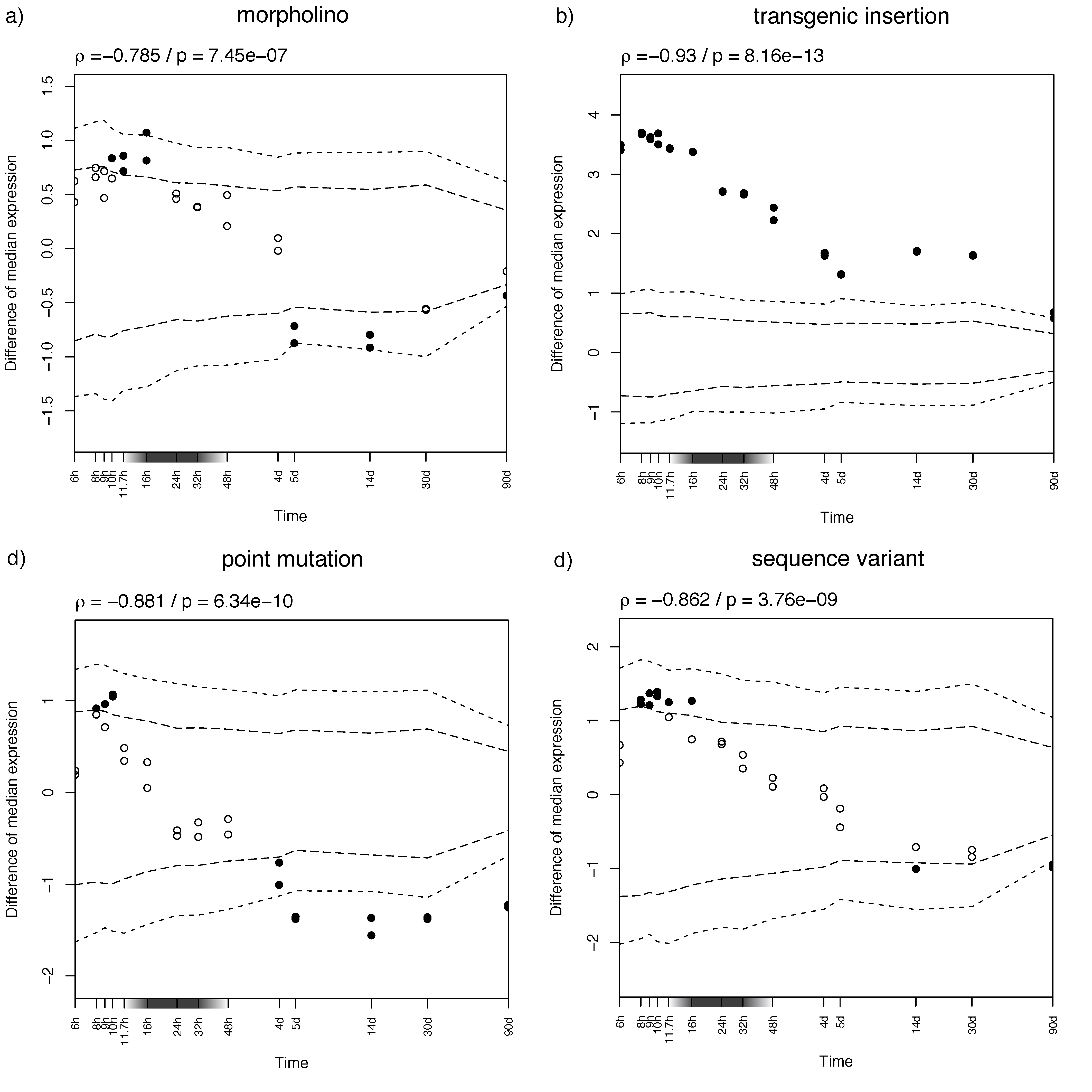

Supplement: Figure S5 — Significance of the expression difference between zebrafish genes inducing abnormal phenotypes when non functional and non-annotated genes for 4 different techniques. These randomization plots refer to Figure S4. Legend as in Figure 2B. (1.05 MB TIF) [file pgen.1000311.s005.tif]
