# Supplementary material for: Developmental Constraints on Vertebrate Genome Evolution
Source: PLoS Genet. 2008 Dec 19;4(12):e1000311. doi: 10.1371/journal.pgen.1000311 (PMC2600815; doi:10.1371/journal.pgen.1000311)
Supplement: Table S1 — Gene Ontology analysis. The two groups analyzed are the genes experiencing an increase of expression along development (late expression, cluster 4) and the genes experiencing a decrease of expression (early expression, cluster 3) (Figure 3). Molecular Function and Biological process ontologies were analyzed with the “elim” algorithm of the Bioconductor package topGO (see Methods). (0.02 MB PDF) [file pgen.1000311.s006.pdf]

Group	Ontology	Direction	GO ID	Term	Annotated	Significant	Expected	p-value	adjusted p-value (FDR)
Early expression	MF	Enriched	GO:0008026	ATP-dependent helicase activity	32	19	9.07	0.00023	0.08671
			GO:0003899	DNA-directed RNA polymerase activity	18	12	5.1	0.0008	0.1046175
			GO:0004926	non-G-protein coupled 7TM receptor activity	8	7	2.27	0.00087	0.1046175
			GO:0008270	zinc ion binding	519	178	147.04	0.00111	0.1046175
			GO:0003702	RNA polymerase II transcription factor activity	19	12	5.38	0.00161	0.121394
		Depleted	GO:0001584	rhodopsin-like receptor activity	73	4	20.68	7.50E-07	0.00028275
			GO:0005509	calcium ion binding	189	32	53.55	0.00015	0.02639
			GO:0005102	receptor binding	105	8	29.75	0.00021	0.02639
			GO:0005262	calcium channel activity	115	17	32.58	0.00043	0.0405275
			GO:0019855	calcium channel inhibitor activity	113	17	32.02	0.00063	0.047502
			GO:0005198	structural molecule activity	187	30	52.98	0.00125	0.078541667
			GO:0015077	monovalent inorganic cation transmembrane transporter activity	54	3	15.3	0.00186	0.0970775
			GO:0004497	monooxygenase activity	54	6	15.3	0.00206	0.0970775
			GO:0004871	signal transducer activity	371	68	105.11	0.00257	0.107654444
			GO:0008135	translation factor activity, nucleic acid binding	36	3	10.2	0.00336	0.126672
			GO:0005179	hormone activity	35	3	9.92	0.00434	0.148743636
			GO:0008083	growth factor activity	40	4	11.33	0.00476	0.149543333
	BP	Enriched	GO:0006396	RNA processing	61	31	17.35	0.00017	0.12971
		Depleted	GO:0007186	G-protein coupled receptor protein signaling pathway	103	7	29.3	3.20E-08	2.44E-05
			GO:0006783	heme biosynthetic process	56	4	15.93	7.60E-05	0.028994
			GO:0006816	calcium ion transport	118	17	33.56	0.00022	0.055953333
			GO:0006118	electron transport	132	21	37.55	0.0005	0.095375
Late expression	MF	Enriched	GO:0005201	extracellular matrix structural constituent	8	8	1.09	1.10E-07	4.15E-05
			GO:0005509	calcium ion binding	189	50	25.73	1.40E-06	0.0002639
			GO:0019855	calcium channel inhibitor activity	113	32	15.39	2.70E-05	0.003393
			GO:0005262	calcium channel activity	115	32	15.66	3.90E-05	0.00367575
			GO:0019842	vitamin binding	20	9	2.72	0.00063	0.047502
		Depleted	GO:0003676	nucleic acid binding	1043	107	142.01	0.00021	0.07917
	BP	Enriched	GO:0006816	calcium ion transport	118	33	15.94	2.20E-05	0.0087745
			GO:0006096	glycolysis	32	16	4.32	2.30E-05	0.0087745
			GO:0030574	collagen catabolic process	19	10	2.57	5.60E-05	0.014242667
			GO:0030239	myofibril assembly	9	6	1.22	0.00035	0.0667625
			GO:0009310	amine catabolic process	10	6	1.35	7.70E-04	0.117502
