# Supplementary material for: Developmental Constraints on Vertebrate Genome Evolution
Source: PLoS Genet. 2008 Dec 19;4(12):e1000311. doi: 10.1371/journal.pgen.1000311 (PMC2600815; doi:10.1371/journal.pgen.1000311)
Supplement: Text S1 — Supplementary text. (0.03 MB DOC) [file pgen.1000311.s009.doc]

## Supplementary results for constraints on gene loss-of-function in zebrafish

Relative to the results for essential genes, we expected similar results for genes inducing phenotypes reported as “abnormal” when non functional. These would be in the large majority of cases counter-selected in nature. As enough data were available for this category of genes, we investigated an eventual methodological bias by plotting separately the main categories of mutants described for zebrafish: transgenic insertion, point mutation, sequence variant, and morpholino knock-down. The median expression profiles are shown in Figure S4, with again non-annotated genes for reference. For abnormal phenotypes reported after a transgenic insertion, genes are globally more expressed than the other categories. This is consistent with observations that this type of mutations is biased towards transcriptionally active genes [1]. For all types of mutants, the pattern is similar to that of essential genes (Figure S5): the largest difference to reference genes is early in development, followed by a progressive decrease of the difference; the minimum is reached for the larval stage. No specific trend is seen around the phylotypic stage.

## Supplementary results for constraints on gene duplication

The trends of Figures 5, 6, S1 and S2, are also apparent using the clusters based on gene expression in zebrafish (Figure 3), with genes whose expression is highest in early development less often kept in duplicate than genes with highest expression in late development, for both the fish specific genome duplication (4.7 % vs. 8.9%) and 2R (4.4 % vs. 5.5%).

Duplicate genes can arise from other sources than whole genome duplications. But we do not observe any significant trends for single gene duplicates in the zebrafish and mouse lineages (not shown). In this case, bias in retention may be masked by other mechanisms, such as bias in generation or fixation of duplicates [2,3].

1. Parinov S, Emelyanov A (2007) Transposable elements in fish functional genomics: technical challenges and perspectives. Genome Biology 8: S6.

2. Davis JC, Petrov DA (2005) Do disparate mechanisms of duplication add similar genes to the genome? Trends Genet 21: 548-551.

3. Vinckenbosch N, Dupanloup I, Kaessmann H (2006) Evolutionary fate of retroposed gene copies in the human genome. Proc Natl Acad Sci USA 103: 3220-3225.
